# Quercetin Affects Hsp70/IRE1*α* Mediated Protection from Death Induced by Endoplasmic Reticulum Stress

**DOI:** 10.1155/2015/645157

**Published:** 2015-04-02

**Authors:** Antonello Storniolo, Marisa Raciti, Alessandra Cucina, Mariano Bizzarri, Livia Di Renzo

**Affiliations:** ^1^Department of Experimental Medicine, Sapienza University, Viale Regina Elena 324, 00161 Rome, Italy; ^2^Department of Surgery P. Valdoni, Sapienza University, Via A. Scarpa 14, 00161 Rome, Italy

## Abstract

Relative to their normal counterparts, tumor cells generally exhibit a greater “stress phenotype” and express heat shock proteins (Hsp) that represent candidate targets for anticancer therapy. Here we investigated the role of Hsp70 in survival induced by endoplasmic reticulum (ER) stressors in human leukemia U937 cells. Quercetin, a major dietary flavonoid, or specific silencing affected the expression level of Hsp70 and did not allow the upregulation of inositol-requiring kinase 1*α* (IRE1*α*), the prototype ER stress sensor regulating the unfolded protein response (UPR), that protects the cells against the stress of misfolded proteins in the ER. The reduction of Hsp70 prevented the upregulation of immunoglobulin heavy-chain binding protein (BiP), but not of CCAAT/enhancer-binding protein-homologous protein (CHOP), and induced apoptosis. Also specific silencing of IRE1*α* or inhibition of its endoribonuclease activity by 4*μ*8c hampered the upregulation of BiP, but not of CHOP, and induced apoptosis. These results suggest that drugs affecting the Hsp70-IRE1*α* axis, like quercetin, or affecting directly IRE1*α* may represent an effective adjuvant antileukemia therapy.

## 1. Introduction

Disturbances in ER calcium homeostasis or protein processing cause the accumulation of misfolded or unfolded proteins in the ER, a cellular condition referred to as “ER stress.” Adaptation to ER stress is mediated by the induction of the unfolded protein response (UPR), a regulated and complex signal transduction pathway transmitting information to the cytosol and nucleus to increase protein folding capacity of the ER [1–3]. However, cells undergo apoptosis when adaptation mechanisms are unable to alleviate the ER stress [4, 5]. Thus, the UPR serves to mitigate the stress or, alternatively, to eliminate stressed cells in order to protect the organism.

The hallmark of the UPR is the upregulation of ER chaperones and folding enzymes, which are required to bind the unfolded proteins and prevent their aggregation [6]. Also a transient attenuation of protein synthesis participates to the UPR by limiting the load of proteins under conditions not well suited to their proper folding [7].

Three resident ER transmembrane sensors detect unfolded proteins in the ER to initiate three distinct UPR branches: inositol-requiring kinase-1*α* (IRE1*α*), activating transcription factor-6 (ATF6), and protein kinase RNA- (PKR-) like ER kinase (PERK) [3, 6–8]. All these sensors have luminal domains that bind BiP under nonstress conditions. Once the ER stress leads to accumulation of unfolded proteins, BiP is released from the UPR sensors and binds to the unfolded proteins. These events trigger the activation of the ER sensors [9, 10]. IRE1*α* is evolutionarily conserved from yeast to humans; it is a Ser/Thr protein kinase and endoribonuclease that, upon activation, initiates the splicing of the mRNA encoding X-box binding protein 1 (XBP-1), converting it into a highly active transcription factor, termed XBP-1s. This is a key regulator of ER folding capacity that upregulates important genes related to control of protein quality, ER translocation, glycosylation, and ER/Golgi biogenesis [11, 12]. XBP-1s regulates several UPR target genes including ER chaperones like BiP, transcription factors like CHOP, and other proteins [13]. BiP plays an antiapoptotic and cytoprotective role in early embryogenesis, oncogenesis, neurodegenerative diseases, and atherosclerosis [13, 14], while CHOP has been linked to apoptosis [15, 16]. CHOP downregulates antiapoptotic proteins like Bcl2 and increases free oxygen species, causing mitochondrial membrane damage and cytochrome C release.

Heat shock proteins (Hsp), classified according to their approximate molecular size, comprise several members exhibiting different expression patterns or expressed in different cell compartments. The Hsp70 family has more than 8 members. It includes the major cytoplasmic forms called heat shock cognate (Hsc) 70, Hsp73, HspA8, Hsp70, the mitochondrial protein GRP75, and the endoplasmic reticulum-resident GRP78. Hsc70 is constitutively and ubiquitously expressed. Hsp70, also named Hsp72, is generally expressed at very low or undetectable levels in unstressed normal cells while it is highly expressed in many malignant tumors, consistent with the idea that it represents a prosurvival factor, playing an essential role as chaperone in protein folding [17–19]. Overexpressed Hsp72 can bind to IRE1*α* and enhance its RNase activity, promoting adaptation to ER stress and cell survival [20]. A role of Hsp70 in tumorigenesis has been suggested, based on the fact that its overexpression in transgenic mice results in T-cell lymphoma [21]. The high expression of Hsp70 in tumor cells increases the resistance to apoptosis and has been associated with poor prognosis of cancer patients [22–25]. An altered expression or function of Hsp70 has been implicated in other human diseases, associated with defects in protein conformation or folding, as disorders caused by mutant proteins or some neurodegenerative diseases, such as Alzheimer's disease and Parkinson's disease [26, 27]. Furthermore, it is known that Hsp70 downregulation results in apoptosis in cancer cells but not in untransformed cells, which makes this protein an attractive target for molecular cancer therapeutics and chemoprevention.

In the present studies we set out to investigate the role of Hsp70, constitutively expressed in human monoblastic leukemia U937 cells, in the regulation of the UPR induced by the ER stressors tunicamycin (TN) and thapsigargin (TG). TN inhibits N-linked glycosylation resulting in accumulation of misfolded proteins, whereas TG inhibits Ca^++^ uptake by the ER, both resulting in ER stress induction. Hsp70 was silenced or its expression was reduced by quercetin (Q) [28], a major dietary flavonoid, that is going to be evaluated in a cancer clinical trial, based on its antiproliferative and anti-tyrosine kinase activities [29].

The present studies demonstrate a functional link between Hsp70 and IRE1*α*: targeting Hsp70 with Q or specific silencing, while not modifying CHOP upregulation, did not allow the upregulation of IRE1*α* and BiP following ER stress and promoted cell death. These results indicate that quercetin, in combination with drugs causing endoplasmic reticulum stress, may represent an effective adjuvant antileukemia therapy.

## 2. Materials and Methods

### 2.1. Materials

Antibody anti-*β*-actin, BSA, FCS, HBSS, l-glutamine, penicillin-streptomycin, PBS, propidium iodide (PI), Q, RNAse, RPMI-1640, and tunicamycin were from Sigma-Aldrich (St. Louis, MO, USA). Antibodies anti-BiP, -caspase 3, -CHOP, -Hsp70, -Hsp90, -IRE1*α*, and -PARP were from Cell Signaling Technology (Beverly, MA, USA). Horseradish peroxidase- (HRP-) conjugated anti-rabbit- and anti-mouse-immunoglobulin antibodies, enhanced chemiluminescence (ECL) reagents, and Hyperfilm-ECL film were from Amersham (Arlington Heights, IL, USA). Lipofectamine RNAiMAX and OPTI-MEM medium, small interfering- (siRNA-) IRE1, and relative scrambled siRNA (Ambion) were from Life Technologies (Invitrogen, San Giuliano Milanese, Italy). 2-Phenylethynesulfonamide (PES) and 4*μ*8c were from Merck (INALCO, Milan, Italy). RC-DC protein assay, SDS-sample buffer, protein standard, SDS-PAGE reagents, and polyvinylidene difluoride (PVDF) membranes were from Bio-Rad Laboratories (Segrate, Italy). Antibody anti-tubulin, siRNA-Hsp70, and scrambled siRNA were purchased from Santa Cruz Biotechnology (Tebu-Bio, Magenta, Italy). Thapsigargin and z-VAD.fmk were from Calbiochem (San Diego, CA, USA). Other reagents were of the highest purity and purchased from Bio-Rad, Invitrogen, or Sigma.

### 2.2. Cell Viability and Growth

The human monoblastic leukemia cell line U937, derived from a patient with diffuse histiocytic lymphoma, was used. Cells were grown in complete medium (RPMI-1640 medium supplemented with 5% heat-inactivated FCS, 2 mM glutamine, 100 units/mL penicillin, and 100 *μ*g/mL streptomycin) at 37°C, in fully humidified 95% room air/5% CO_2_. Cells were resuspended three times a week in fresh complete medium to 3 × 10^5^/mL. Cell growth was evaluated by hemocytometry counts of cells excluding trypan blue (0.04% Trypan blue in PBS, w/v), and viability was assessed by calculating alive (trypan blue excluding) cells as percentage of all cells counted (trypan blue excluding and not excluding). Cells used in every experiment were ≥94% viable and taken from cultures in exponential growth. They were washed once and resuspended in complete medium, 1 × 10^6^/mL, and transferred to multiwell plates. They were then treated with inhibitors or vehicles, incubated for 30 min, and subsequently exposed to test agents or, again, to vehicles. At the end of each experiment, the cells were gently mixed and aliquots were taken for cell counting, PI staining, and cell cycle analysis. The vehicles, even when used in combination, were ≤0.3% (v/v) and did not modify any investigated parameter in comparison with control cultures.

### 2.3. Flow Cytometry Analysis of Cell Death

Nuclear DNA fragmentation was quantified by flow cytometry of hypodiploic (subG1) DNA after cell fixation and PI staining [30, 31]. Briefly, cells were washed with PBS, pelleted and fixed in ice cold ethanol/water (70/30, v/v) for 1 h, pelleted again and washed twice with PBS, and finally resuspended in PBS containing RNAse (20 *μ*g/mL) and PI (100 *μ*g/mL). Events in the different cell cycle phases were gated manually using an EPICS XL cytofluorimeter (Beckman Coulter, Hialeah, Fl, USA). At least 10.000 events/sample were acquired. Collected data were analyzed using the Multicycle software for DNA content and cell cycle analysis (Phoenix Flow System, San Diego, CA, USA). The subG1 events representative of the apoptotic cells, and the events in the other cell cycle phases, are given as percentage of the total cell population.

Membrane permeability, indicative of cell death, was investigated by resuspending the cells in HBSS containing PI (200 *μ*g/mL) at room temperature and analyzed by flow cytometry (EPICS-XL), measuring the fluorescence emission at >575 nm (FL3).

### 2.4. Western Blot Analysis

Whole cell lysates were prepared as previously described [32]. Briefly, the cells were kept for 30 min on ice in lysis buffer (NaCl 150 mM, CaCl_2_ 1 mM, MgCl_2_ 1 mM, NaN_3_ 0.1%, NaF 10 mM, Triton X-100 1% (v/v), orthovanadate 1 mM, aprotinin 2 *μ*g/mL, leupeptin 2 *μ*g/mL, iodoacetamide 10 mM, PMSF 2 mM, and pepstatin 20 *μ*M). The appropriate volumes of 4x SDS-sample buffer (v/v) were then added. Cell lysates were briefly sonicated, warmed at 95°C for 5 min, and cleared by 14000 ×g centrifugation in a microfuge for 15 min at 4°C. Supernatants were collected and proteins were quantified by RC DC protein assay. Equal amounts of proteins were separated from the different samples by SDS-PAGE, and blotted onto PVDF membranes. Transfer efficiency was checked with Ponceau staining. The blots were blocked in tris-buffered saline (TBS) containing BSA 5% (w/v), probed with specific primary antibodies (anti-BiP, -CHOP, -Hsp70, -IRE1*α*, or -PARP), washed with PBS-Tween 20, and then incubated with the appropriate peroxidase-conjugated secondary antibody. Finally, in order to control protein loading, each membrane was probed to detect *β*-actin, tubulin, Hsp90, or caspase 3 and the appropriate peroxidase-conjugated secondary antibody. For each antibody were used the dilutions and incubation times suggested by the manufacturer. Immunodetection was performed using the ECL reagents and Hyperfilm-ECL film. Densitometry quantitation of the bands was performed using ImageJ software (National Institutes of Health, Bethesda, MD, USA) on a Mac OS X (Apple Computer International, Cupertino, CA, USA).

### 2.5. siRNA

RNA knockdown was performed with pools of siRNA duplexes. Briefly, cells were washed and resuspended in OPTI-MEM medium, transfected with siRNA specific for human Hsp70 and scrambled siRNA (Santa-Cruz) or with siRNA specific for IRE1*α* and relative scrambled siRNA (Ambion), using Lipofectamine RNAiMAX according to the manufacturer's guidelines and as we previously described [33]. After 12 h of incubation, RPMI 1640 containing 20% fetal calf serum was added without removing the transfection medium. The cells were cultured for further 60 h. After centrifugation, the medium was replaced with fresh RPMI-1640, containing 10% fetal calf serum, and the cells cultured again in the presence or not of TN.

### 2.6. Statistical Analysis

Results are expressed as the mean ± standard deviation (SD) of repeated experiments, as indicated in the figure legends. Statistical differences between the data sets were evaluated using unpaired, two-tailed Student's *t*-test. Values of *P* < 0.05 were considered statistically significant.

## 3. Results 

### 3.1. Survival of U937 Cells under Moderate ER Stress Conditions

**Figure 1:**
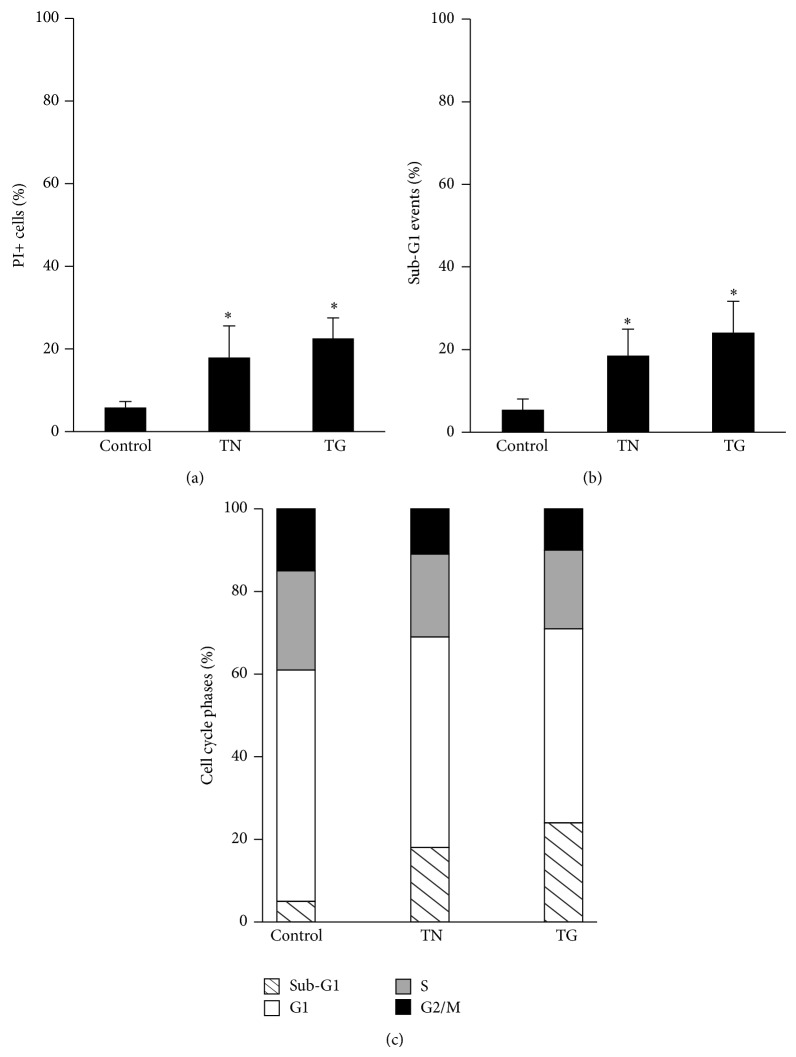
Low doses of the ER stressors, TN or TG, are not cytotoxic to U937 cells. U937 cells either exposed or not to TN 1 *μ*M or TG 200 nM for 24 h. (a) A cell portion was unfixed, stained with PI and immediately ≥10000 events were analyzed in a cytofluorimeter in order to evaluate the dead cells incorporating PI as percentage of total events. (b and c) A cell portion was fixed and thereafter stained with PI in order to evaluate by cytofluorimetry, among ≥10000 events, those in sub-G1 (b) and in sub-G1, G1, S, and G2M (c) of the cell cycle. The reported values represent the means and error bars, the S.D. of dead cells (PI+), or sub-G1 events of 12 independent experiments. Assessment of cell death showed statistically significant differences between the data obtained in the cultures treated with TN or TG in comparison with the untreated cultures (^∗^ *P* < 0.0001).

TN and TG are ER stressors that induce U937 cell death in a dose-dependent manner and that activate a prosurvival pathway at low concentrations [33]. In particular, TN (1 *μ*M) caused a moderate increase of PI incorporating (PI+) cells (18 ± 7%) in comparison with untreated cultures (6 ± 1%) (Figure 1(a)), in addition to the appearance of 19 ± 6% of sub-G1 events (Figure 1(b)). Similarly, TG (200 nM) caused a fall in cell viability (PI+ cells 23 ± 5%) (Figure 1(a)) and induced the appearance of sub-G1 events (24 ± 7%) (Figure 1(b)). Sub-G1 events were studied by cytofluorimetry of cell cycle phases of cells fixed and stained with PI. The hypodiploid DNA events were easily discernible from the narrow peak of cells with diploid DNA content; they are considered to be indicative of apoptotic nuclei [30, 33]. Furthermore, analysis of events in the different cell cycle phases showed that TN (1 *μ*M) and TG (200 nM) caused a decrease in S and G2M phases, while the percentage of G1 events were apparently unchanged (Figure 1(c)). Cell counts indicated that neither TN nor TG allowed cell growth (not shown). These results show that these ER stressors, at the indicated low concentrations, cause activation of a prosurvival pathway in U937 cells, arrested in G1 cell cycle phase. To investigate this prosurvival pathway, all the experiments were performed with TN 1 *μ*M and TG 200 nM, and viability parameters were investigated after 24 h.

### 3.2. Quercetin Downregulates the Expression of Hsp70 in U937 Cells

**Figure 2:**
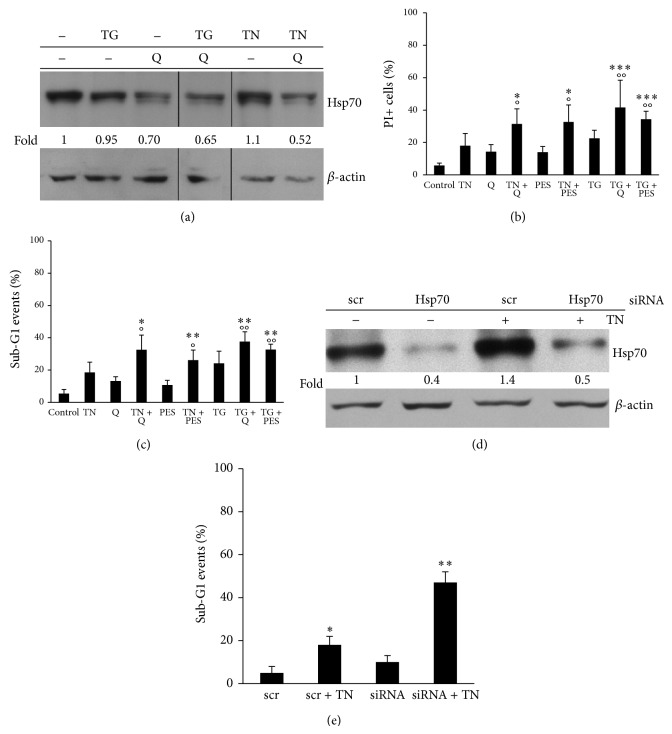
Hsp70 promotes survival of ER stressed cells. (a) Hsp70 was detected by western blot in the lysates of U937 cells either exposed or not to Q (10 *μ*M, 30 min) and thereafter to none or TN 1 *μ*M or TG 200 nM for 24 h. Blotted proteins were probed with anti-Hsp70 followed by peroxidase-conjugated secondary antibody. Western blot of *β*-actin is shown at the bottom as a loading control. Representative blots are shown. Densitometric quantification of the bands is shown at the bottom of the relevant lines as the ratio of Hsp70 in each line to the Hsp70 value observed in the lysate of untreated U937 cells. (b and c) Detection of cell death by evaluation of PI+ cells (b) or of sub-G1 events (c). U937 cells were pretreated for 30 min with none or Q (10 *μ*M) or PES (10 *μ*M) and then with none or TN (1 *μ*M) or TG (200 nM). A portion of cells were unfixed and stained with PI (b) or fixed and stained with PI to evaluate sub-G1 events in the cell cycle (c) under cytofluorimetry. Both types of investigation were performed on ≥10000 events. The values reported are means ± S.D. (*n* = 6). Assessment of cell death showed statistically significant differences between the data obtained in the cultures treated with TN or TG together with Q or with PES, in comparison with the cultures treated only with Q (°*P* < 0.0001) or only with PES (°°*P* < 0.0001) or only with TN or TG (^∗^ *P* < 0.0001; ^∗∗^ *P* < 0.003; ^∗∗∗^ *P* < 0.03). (d) Western blot analysis of Hsp70 by specific antibody in the lysates of U937 cells transfected with equal amounts of Hsp70 siRNA or scr-siRNA for 72 h and exposed to none or to TN 1 *μ*M for further 24 h. Western blot analysis of *β*-actin is shown at the bottom, as a loading control and also to confirm the specificity of the transfected siRNA. Representative blots are shown. Densitometric quantification of the bands is shown at the bottom of the relevant lines as the ratio of Hsp70 in each line to the Hsp70 value observed in the lysate of untreated U937 cells transfected with scrambled siRNA. (e) U937 cells transfected with Hsp70 siRNA or scr-siRNA for 72 h and exposed or not to TN 1 *μ*M for 24 h were fixed with ethanol, stained with PI, and analyzed under cytofluorimetry to detect sub-G1 events in the cell cycle. Here are shown the mean ± S.D. of four independent experiments. Assessment of cell death showed statistically significant differences between the data obtained in the cultures treated with scr-siRNA and TN in comparison with the cultures treated only with scr-siRNA (^∗^ *P* < 0.005) or with Hsp70 siRNA and TN in comparison with scr-siRNA or Hsp70 siRNA (^∗∗^ *P* = 0.001).

Hsp70 is present at constitutively high levels in various human tumors, in contrast to its low expression in unstressed normal cells. It is an important chaperone that plays a key role in conformational maturation and stabilization of proteins involved in cell growth and survival. Also U937 tumor cells express constitutively rather high levels of Hsp70, not apparently modified by the ER stress induced by TN or TG (Figure 2(a)). Q is known to inhibit the synthesis of Hsp70 in some human cancer cell lines [28]. This flavonoid, at high doses, is cytotoxic and can block proliferation of U937 cells (unpublished results). Thus, we used the concentration 10 *μ*M to avoid its cytotoxic effects. We found that quercetin (30 min, cell pretreatment) reduced the expression of Hsp70 in U937 cells under basal conditions as well as upon TN or TG treatments (Figure 2(a)).

### 3.3. Hsp70 Protects ER Stressed Cells from Death

Q caused per se a moderate increase of U937 cell death, as determined by evaluation of PI+ cells (14 ± 4%) and sub-G1 events (13 ± 3%) (Figures 2(b) and 2(c)). However, when combined with TN or TG, it caused a conspicuous increase of PI+ cells and sub-G1 events (Figures 2(b) and 2(c)). Thus, Q turns the survival induced by the ER stressors into cell death. PES is a pharmacological inhibitor of Hsp70 [34, 35]. It (10 *μ*M, 30 min cell pretreatment) was slightly cytotoxic for U937 cells (PI+ cells 14 ± 3%; sub-G1 events 10.7 ± 3%) and more cytotoxic when associated with TN or TG (Figures 2(b) and 2(c)). In order to rule out off-target effects of the pharmacological inhibitors, Hsp70 expression was silenced by specific siRNA. After transfection time (72 h), TN was added for the subsequent 24 h. In comparison with scr-siRNA, Hsp70 specific silencing brought about an effective reduction of Hsp70 (Figure 2(d)) and caused a strong increase of cell death in ER stressed cells (Figure 2(e)).

These results show that Hsp70 promotes survival of ER stressed cells.

### 3.4. Hsp70 and IRE1*α* Are Functionally Linked

**Figure 3:**
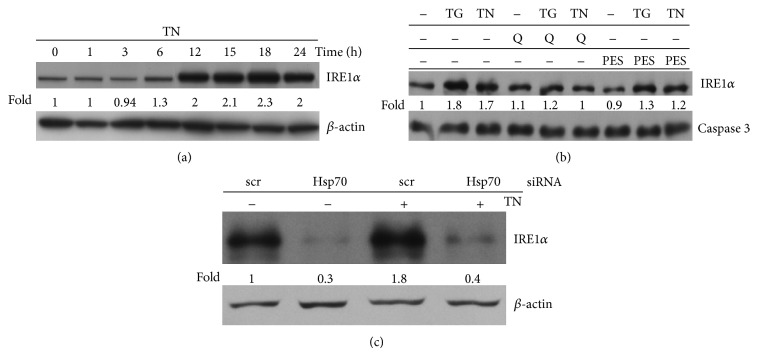
IRE1*α* expression is linked to Hsp70. (a) Western blot was performed with anti-IRE1*α* followed by peroxidase-conjugated secondary antibody to analyze this ER sensor in the lysates of untreated (0 h) or TN treated U937 cells for the hours indicated. Western blot of *β*-actin is shown at the bottom as a loading control. Representative blots are shown. Densitometric quantification of the bands is shown at the bottom of the relevant lines as the ratio of IRE1*α* in each line to the IRE1*α* value observed in the lysate of untreated U937 cells. (b) Western blot was performed with anti-IRE1*α* followed by peroxidase-conjugated secondary antibody to analyze this ER sensor in the lysates of untreated cells or Q (10 *μ*M) or PES (10 *μ*M) pretreated cells, thereafter treated with none or TN or TG for 24 h. Western blot of caspase 3 is shown at the bottom as a loading control. Representative blots are shown. Densitometric quantification of the bands is shown at the bottom of the relevant lines as the ratio of IRE1*α* in each line to the IRE1*α* value observed in the lysate of untreated U937 cells. (c) Western blot analysis of IRE1*α* by specific antibody in the lysates of U937 cells transfected with equal amounts of Hsp70 siRNA or scr-siRNA (Santa Cruz) for 72 h and exposed to none or to TN 1 *μ*M for further 24 h. Western blot analysis of *β*-actin is shown at the bottom, as a loading control and also to confirm the specificity of the transfected siRNA. Representative blots are shown. Densitometric quantification of the bands is shown at the bottom of the relevant lines as the ratio of IRE1*α* in each line to the IRE1*α* value observed in the lysate of untreated U937 cells transfected with scrambled siRNA.

Among the three major transmembrane sensors of ER stress (IRE1*α*, PERK, and ATF6), IRE1*α* is the prototype ER stress sensor, evolutionarily conserved from yeast to human. The cytoprotective output of IRE1*α* is present across all eukaryotes. We examined the expression level of this sensor in U937 cells treated with TN and found that it was upregulated after 6 h, even more after 12, 15, and 18 h, and it remained high after 24 h (Figure 3(a)). Similar results were obtained using TG, as ER stressor (not shown).

In order to investigate the link between Hsp70 and IRE1*α*, U937 cells were pretreated with Q or PES, as the former drug affects Hsp70 expression, while the latter inhibits it. Both pretreatments prevented the upregulation of IRE1*α* following ER stress (Figure 3(b)). To rule out any unspecific effect of the used inhibitors, IRE1*α* was analyzed by western blot in cells silenced for Hsp70. In comparison with scr-siRNA, Hsp70 silencing effectively decreased the expression of constitutive IRE1*α* and prevented the increased expression of this ER sensor caused by TN treatment (Figure 3(c)).

These experiments indicate a functional link between Hsp70 and IRE1*α*.

### 3.5. Hsp70 Regulates BiP Expression through IRE1*α*

**Figure 4:**
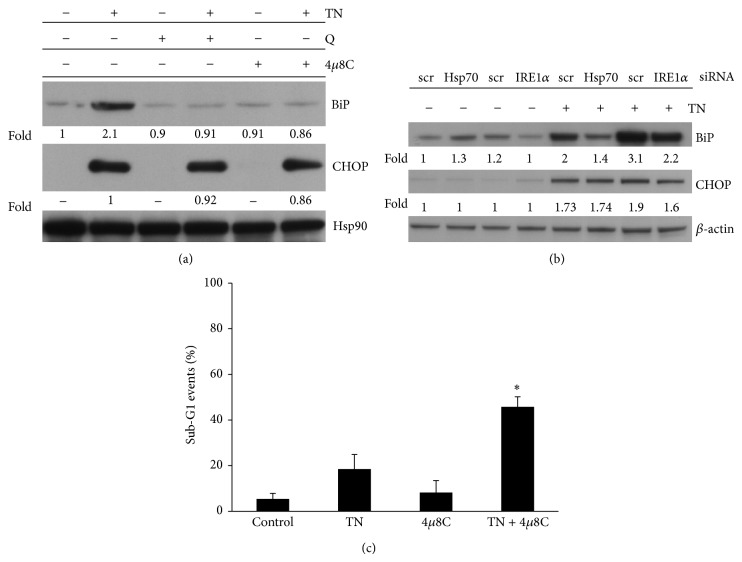
Impairment of Hsp70 prevents upregulation of BiP but not of CHOP. (a) Western blot was performed with anti-BiP or anti-CHOP followed by peroxidase-conjugated specific secondary antibody to analyze these proteins in the lysates of untreated cells or Q (10 *μ*M) or 4*μ*8c (10 *μ*M) pretreated cells and thereafter treated with none or TN for 24 h. Western blot of Hsp90 is shown at the bottom as a loading control. Representative blots are shown. Densitometric quantification of the bands is shown at the bottom of the relevant lines as the ratio of BiP or CHOP in each line, respectively, to the BiP or CHOP values observed in the lysate of untreated U937 cells or to the CHOP value observed in TN treated cells. Western blot analysis of Hsp90 is shown at the bottom, as a loading control. (b) Western blot analysis of BiP or CHOP by specific antibody in the lysates of U937 cells transfected with equal amounts of Hsp70 siRNA or scr-siRNA (Santa Cruz) or equal amounts of IRE1*α* siRNA or scr-siRNA (Ambion) for 72 h and exposed to none or to TN 1 *μ*M for further 24 h. Western blot analysis of *β*-actin is shown at the bottom, as a loading control and also to confirm the specificity of the transfected siRNA. Representative blots are shown. Densitometric quantification of the bands is shown at the bottom of the relevant lines as the ratio of BiP or CHOP in each line, respectively, to the BiP or CHOP values observed in the lysate of untreated U937 cells transfected with scrambled siRNA (Santa Cruz). (c) Detection of cell death by evaluation of sub-G1 events. U937 cells were pretreated for 30 min with none or 4*μ*8c (10 *μ*M) and thereafter for 24 h with none or TN (1 *μ*M). The cells were fixed and stained with PI to evaluate sub-G1 events in the cell cycle by cytofluorimetric analysis performed on ≥10000 events. The values reported are means ± S.D. (*n* = 5). Assessment of cell death showed statistically significant differences between the data obtained in the cultures treated with TN and 4*μ*8c in comparison with the cultures treated only with TN (^∗^ *P* < 0.0001).

A number of studies have shown that cell survival or death decisions during the UPR are, respectively, mediated via the antiapoptotic BiP or the proapoptotic CHOP. We therefore examined the expression of these proteins by western blot in ER stressed U937 cells. TN, as previously reported, resulted in upregulation of BiP and CHOP, which became evident after 6 h and increased after 12 and 24 h of treatment [33]; quercetin or specific silencing of Hsp70 did not influence the expression of CHOP, while they prevented substantially BiP upregulation (Figures 4(a) and 4(b)).

**Figure 5:**
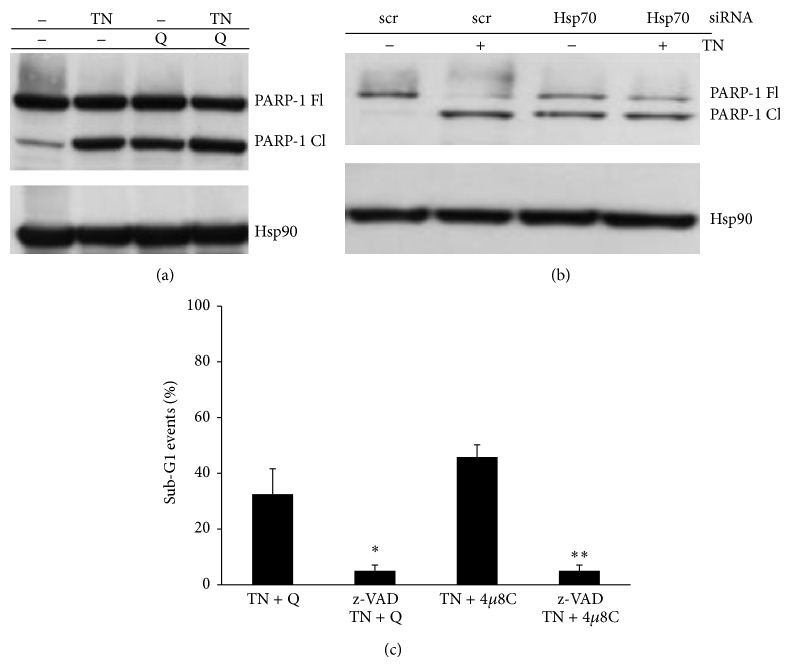
Hsp70-IRE1*α* axis protects from apoptosis. (a) Q induces fragmentation of PARP-1. Western blot was performed with anti-PARP-1 followed by peroxidase-conjugated secondary antibody to analyze this protein (FL = full length; CL = cleaved) in the lysates of untreated or Q (10 *μ*M) pretreated cells and thereafter treated with none or TN for 24 h. Western blot of Hsp90 is shown at the bottom as a loading control. Representative blots are shown. (b) Western blot analysis of PARP-1 (FL = full length; CL = cleaved) by specific antibody in the lysates of U937 cells transfected with equal amounts of Hsp70 siRNA or scr-siRNA (Santa Cruz) for 72 h and exposed to none or to TN 1 *μ*M for further 24 h. Western blot analysis of Hsp90 is shown at the bottom, as a loading control and also to confirm the specificity of the transfected siRNA. Representative blots are shown. (c) Detection of cell death by evaluation of sub-G1 events. U937 cells were pretreated for 60 min with none or z-VAD.fmk (70 *μ*M), then for further 30 min with none or Q (10 *μ*M) or 4*μ*8c (10 *μ*M), and thereafter for 24 h with none or TN (1 *μ*M). The cells were fixed and stained with PI to evaluate sub-G1 events in the cell cycle by cytofluorimetric analysis performed on ≥10000 events. The values reported are means ± S.D. (*n* = 5). Assessment of cell death showed statistically significant differences between the data obtained in the cultures treated with TN and Q and z-VAD in comparison with the cultures treated only with TN and Q (^∗^ *P* < 0.0001) or between the data obtained in the cultures treated with TN and 4*μ*8c and z-VAD in comparison with the cultures treated only with TN and 4*μ*8c (^∗∗^ *P* < 0.0001).

IRE1*α* is a Ser/Thr protein kinase and endoribonuclease that, upon activation, initiates the unconventional splicing of the X-box binding protein (XBP-1) mRNA. The product of this splicing is XBP-1s, a highly active transcription factor and one of the key regulators of ER folding capacity. 4*μ*8c is an inhibitor of IRE1*α* endoribonuclease activity [36]. IRE1*α* inhibition with 4*μ*8c (10 *μ*M, cell pretreatment 30 min) or its specific silencing prevented BiP, but not CHOP induction after 24 h of TN treatment (Figures 4(a) and 4(b)). When TG was used, similar results were observed (not shown). The inhibition of IRE1*α* by 4*μ*8c caused a slight increase of sub-G1 events, more evident after TN treatment (Figure 5(c)).

These results indicate that in U937 cells undergoing a moderate ER stress Hsp70 promotes BiP upregulation through IRE1*α* activity and that this ER sensor plays a prosurvival role.

### 3.6. Hsp70-IRE1*α* Axis Contrasts Apoptosis in ER Stressed Cells

TN and TG induce apoptosis in U937 cells as above and previously reported [33]. Here we show that in these cells PARP-1 was cleaved, indicating caspase 3 activation (Figure 5(a)). Furthermore, conditions affecting Hsp70 expression, that is, the use of Q or specific silencing of Hsp70, increased PARP-1 cleavage, which appeared more conspicuous after cell treatment with TN (Figures 5(a) and 5(b)).

Detection by flow cytometry of sub-G1 events is considered to be indicative of apoptosis. An increase of sub-G1 events was observed after Hsp70 impairment with Q or after IRE1*α* inhibition with 4*μ*8c that was prevented by the use of z-VAD.fmk, a pan-caspase inhibitor (70 *μ*M, pretreatment for 60 min) (Figure 5(c)).

These results indicate that either the impairment of Hsp70 expression or the inhibition of IRE1*α* endoribonuclease activity cause apoptosis of ER stressed U937 cells. Taken together, these findings suggest that Hsp70-IRE1*α* axis is a kind of hub around which are regulated survival and apoptosis mechanisms.

## 4. Discussion 

ER stress-activated gene transcription is mediated by three different but interconnected pathways: PERK-ATF-4, ATF6, and IRE1*α*-XBP-1s. The transducers of these pathways (PERK, ATF6, and IRE1*α*) sense the presence of unfolded proteins in the ER lumen and convey this information to the nucleus. IRE1*α* activation leads to XBP-1 alternative splicing and, among other proteins, to BiP upregulation involved in cell survival [9–13]. It is known that the knocking down of IRE1*α* enhances cell death under conditions of chronic ER stress and that prolonging of IRE1*α* signaling, independently of ER stress, can promote cell survival [37–39].

A functional crosstalk between Hsp70 and ER stress has been observed in a number of physiological and pathological conditions, such as a cellular model of Parkinson's disease induced by 6-hydroxy dopamine (6-OHDA) or following proteasome inhibition [40, 41]. It has also been reported that the regulation of the UPR is associated with the formation of a stable complex between Hsp72 and the cytosolic domain of IRE1*α* and that Hsp72, although having no effect on the half-life of this ER sensor, enhances its RNase activity, suggesting a direct interaction [20]. Overall, those results have shown that Hsp72 is a component of the UPRosome that, by binding IRE1*α*, enhances XBP-1 signaling and promotes adaptation to ER stress and cell survival.

In the present work we set out to explore the relationship between Hsp70 expressed by U937 leukemia cells and IRE1*α*, in order to test the hypothesis that constitutive Hsp70, enhancing the amplitude of IRE1*α* signaling, promotes cell survival under conditions of ER stress. To this end, we used doses of ER stressors causing a moderate ER stress allowing the survival of U937 cells and we inhibited Hsp70 expression by Q or specific siRNA.

The induction of the Hsp in higher organisms is regulated at the transcriptional and translational levels. The transcription of heat shock genes is regulated by the* cis*-acting heat shock element (HSE) in the promoter region and the* trans*-acting heat shock factor (HSF). Q action has been examined on the promoter region of the human* Hsp70* gene, especially on the formation of the HSF-HSE complex after activation either in vivo by heat shock or in vitro by heat treatment, urea, or Nonidet P-40 [28]. It was detected that Q inhibits the binding of HSF to the HSE both in vivo and in vitro. Thus, Q interaction with HSF leads to the inhibition of this transcription factor and of Hsp70 [28]. Our studies have confirmed that this flavonoid affects Hsp70 expression also in human U937 cells under basal conditions and upon ER stress.

Q or specific silencing of Hsp70 prevented also the upregulation of IRE1*α* induced by ER stress, suggesting a functional link between Hsp70 and IRE1*α*, allowing the increased expression of the latter protein under conditions of ER stress that could be due to an increased translation of IRE1*α* or to a physical connection leading to an increased half-life of IRE1*α*. Although we did not explore the link of Hsp70 to the UPRosome, we were able to pinpoint that, under ER stress, the Hsp70-IRE1*α* axis permits the upregulation of BiP, while it is not affecting CHOP in U937 cells. In fact, the ER stressors induced both BiP and CHOP, while IRE1*α* downregulation prevented only the expression of BiP. However, the cells survived when both BiP and CHOP expression increased, while apoptosis was induced after inhibition of the Hsp70-IRE1*α* axis and BiP downregulation. The same effect on cell survival was obtained also after specific silencing of IRE1*α* or inhibition of its endoribonuclease activity with 4*μ*8c. Thus, Hsp70, by regulating IRE1*α* expression, plays a role in a survival pathway featuring BiP, since this protein is antiapoptotic and prevents ER stress-induced cell death [13, 14].

The accumulation of unfolded proteins following ER stress, in defect of IRE1*α*, is supposed to lead to the activation of the other two sensors [42]. In this context the concomitant decrease of BiP may represent a crucial step, as this protein is an inhibitory chaperone of the three ER sensors. Thus, the possible activation of PERK, via ATF4, may cause the increased expression of the proapoptotic CHOP. As this scenario generally occurs in severe ER stress [42, 43], we hypothesize that the impairment of the Hsp70-IRE1*α* axis turns a mild ER stress into a severe ER stress, associated with increased expression of CHOP.

In conclusion, in this study we show that quercetin, a major dietary flavonoid, downregulates the expression of Hsp70 in monoblastic leukemia U937 cells. Quercetin or specific Hsp70 siRNA prevents the upregulation of IRE1*α* and BiP, during endoplasmic reticulum stress, induced by TN or TG. This condition of ER stress and dysregulation of IRE1*α* and BiP promotes cell death.

These results indicate that, during ER stress, Hsp70 and IRE1*α* represent candidate targets to kill leukemia cells and that quercetin may be employed at this purpose.

## Abbreviations

ATF6: Activating transcription factor 6
BiP: Immunoglobulin heavy-chain binding protein
BSA: Bovine serum albumin
CHOP: CCAAT/enhancer-binding protein-homologous protein
ECL: Enhanced chemiluminescence
ER: Endoplasmic reticulum
FCS: Fetal calf serum
GRP78: 78 kDa glucose-regulated protein
HRP: Horseradish peroxidase
Hsp: Heat shock protein
IRE1*α*: Inositol-requiring kinase 1*α*
PBS: Phosphate buffered saline
PERK: Protein kinase RNA- (PKR-) like ER kinase
PES: 2-Phenylethynesulfonamide
PI: Propidium iodide
PVDF: Polyvinylidene difluoride
Q: Quercetin
RC-DC protein assay: Reducing agent and detergent compatible protein assay
SDS-PAGE: Sodium-dodecyl-sulphate-polyacrylamide gel electrophoresis
siRNA: Small interfering RNA
TBS: Tris-buffered saline
TG: Thapsigargin
TN: Tunicamycin
UPR: Unfolded protein response
XBP-1: X-box binding protein 1.
